# Patient-derived epithelial cell organoids mimic the phenotypic complexity of endometriosis subtypes

**DOI:** 10.1093/humrep/deaf230

**Published:** 2025-11-27

**Authors:** K Gunther, D Liu, M Cortesi, E Powell, E Nesbitt-Hawes, J A Abbott, C E Ford

**Affiliations:** Gynaecological Cancer Research Group, Lowy Cancer Research Centre, School of Clinical Medicine, Faculty of Medicine & Health, UNSW Sydney, Sydney, New South Wales, Australia; Gynaecological Research and Clinical Evaluation (GRACE) Unit, UNSW Sydney, Sydney, New South Wales, Australia; Discipline of Women’s Health, School of Clinical Medicine, UNSW Sydney, Sydney, New South Wales, Australia; National Endometriosis Clinical and Scientific Trials (NECST) Network, UNSW Sydney, Sydney, New South Wales, Australia; Gynaecological Cancer Research Group, Lowy Cancer Research Centre, School of Clinical Medicine, Faculty of Medicine & Health, UNSW Sydney, Sydney, New South Wales, Australia; Discipline of Women’s Health, School of Clinical Medicine, UNSW Sydney, Sydney, New South Wales, Australia; Gynaecological Cancer Research Group, Lowy Cancer Research Centre, School of Clinical Medicine, Faculty of Medicine & Health, UNSW Sydney, Sydney, New South Wales, Australia; Discipline of Women’s Health, School of Clinical Medicine, UNSW Sydney, Sydney, New South Wales, Australia; Laboratory of Cellular and Molecular Engineering “S. Cavalcanti”, Department of Electrical Electronic and Information Engineering “G. Marconi”, University of Bologna, Bologna, Italy; Gynaecological Cancer Research Group, Lowy Cancer Research Centre, School of Clinical Medicine, Faculty of Medicine & Health, UNSW Sydney, Sydney, New South Wales, Australia; Discipline of Women’s Health, School of Clinical Medicine, UNSW Sydney, Sydney, New South Wales, Australia; Gynaecological Research and Clinical Evaluation (GRACE) Unit, UNSW Sydney, Sydney, New South Wales, Australia; Discipline of Women’s Health, School of Clinical Medicine, UNSW Sydney, Sydney, New South Wales, Australia; Prince of Wales Private Hospital, Sydney, New South Wales, Australia; Gynaecological Research and Clinical Evaluation (GRACE) Unit, UNSW Sydney, Sydney, New South Wales, Australia; Discipline of Women’s Health, School of Clinical Medicine, UNSW Sydney, Sydney, New South Wales, Australia; National Endometriosis Clinical and Scientific Trials (NECST) Network, UNSW Sydney, Sydney, New South Wales, Australia; Prince of Wales Private Hospital, Sydney, New South Wales, Australia; Gynaecological Cancer Research Group, Lowy Cancer Research Centre, School of Clinical Medicine, Faculty of Medicine & Health, UNSW Sydney, Sydney, New South Wales, Australia; Discipline of Women’s Health, School of Clinical Medicine, UNSW Sydney, Sydney, New South Wales, Australia

**Keywords:** endometriosis, organoid, *in vitro* model, disease models, preclinical, epithelial cells

## Abstract

**STUDY QUESTION:**

Can patient-derived organoid models be reliably established from diverse surgical phenotypes of endometriosis, and how do clinical factors such as hormonal treatment affect their growth success and morphology?

**SUMMARY ANSWER:**

Endometriosis organoids can be established across all major surgical phenotypes with variable efficiency, and hormonal treatment at the time of biospecimen collection significantly reduces organoid establishment success.

**WHAT IS KNOWN ALREADY:**

Organoid cultures have been developed from eutopic endometrium and select endometriosis tissue biospecimens previously, but their feasibility as pre-clinical models of endometriosis across diverse tissue types and clinical presentations remains unclear.

**STUDY DESIGN, SIZE, DURATION:**

Twenty-eight endometriosis tissue biospecimens were obtained from 23 patients undergoing surgery, with organoid cultures assessed through successive stages of establishment, passage, and cryopreservation.

**PARTICIPANTS/MATERIALS, SETTING, METHODS:**

Endometriosis biospecimens, including deep infiltrating endometriosis (DIE), ovarian endometrioma (OMA), and superficial peritoneal (SUP) biospecimens, were processed into organoid cultures using a validated low-Wnt culture system. Organoid viability, morphology, hormone receptor expression, and cellular composition were evaluated by microscopy, immunohistochemistry, and quantitative morphometric analysis.

**MAIN RESULTS AND THE ROLE OF CHANCE:**

Overall, 22/28 (78.6%) biospecimens established 3-dimensional structures, with 15/28 (53.6%) remaining viable after cryopreservation. Establishment success differed by phenotype (OMA 71.4%, DIE 63.6%, SUP 30%). Progesterone receptor expression was retained in SUP and DIE-derived organoids (7/7, 100%), while OMA-derived organoids showed substantial reductions (4/5 cases). Biospecimens from patients receiving hormonal treatment were smaller (*P* = 0.038) and had reduced organoid establishment success (3/13, 23.1% vs 12/15, 80.0%, *P* = 0.003). Organoids exhibited distinct morphological patterns correlating with disease phenotype.

**LIMITATIONS, REASONS FOR CAUTION:**

Uniform culture conditions may limit growth of certain subtypes, and the *in vitro* organoid models may not fully represent *in vivo* tissue complexity. Sample sizes were modest, and pooling tissues from the same patient could mask intra-patient heterogeneity.

**WIDER IMPLICATIONS OF THE FINDINGS:**

These organoid models offer a promising platform for studying subtype-specific endometriosis biology, including hormone resistance mechanisms, and could inform personalized therapeutic development. The impact of hormonal treatment on organoid viability underscores the need to consider clinical context in pre-clinical models of endometriosis.

**STUDY FUNDING/COMPETING INTEREST(S):**

This work was supported by the National Endometriosis Clinical and Scientific Trials (NECST) Network, funded by the Australian Government Department of Health and Aged Care (Grant 4-I66SNMA), and by a research grant from Endometriosis Australia to C.E.F., D.L., and J.A.A. K.G. is supported by an Australian Government Research Training Program Scholarship and a NECST Network Top-Up Scholarship, which did not influence the conduct or outcomes of this study. The funders had no role in study design, data collection and analysis, decision to publish, or preparation of the manuscript. J.A.A. has received consulting fees from Hologic, Gedeon Richter, and BD, personal payments from Hologic, Bayer, Organon, and Gedeon Richter, travel support from Gedeon Richter, and participated on data safety monitoring advisory boards for Hologic and Gideon Richter. He was the former chair of the Australian Endometriosis Guideline Committee and is the Co-Editor-in-Chief of the Journal of Minimally Invasive Gynaecology. All other authors declare no competing interests.

**TRIAL REGISTRATION NUMBER:**

N/A.

## Introduction

Endometriosis is a chronic, clinically heterogeneous inflammatory condition defined by the presence of endometrium-like lesions outside the uterine cavity. Affecting approximately 190 million people worldwide, endometriosis is associated with significant morbidity including chronic pelvic pain, infertility, and reduced quality of life (World Health Organization, 2023). Despite its prevalence, the pathogenesis of endometriosis remains incompletely understood, and treatment options remain limited to hormonal suppression or surgery, neither of which provides a permanent cure (Alonso *et al.*, 2024).

Endometriosis is commonly classified into three major phenotypes based on lesion appearance and depth during surgery: superficial endometriosis (SUP), deep infiltrating endometriosis (DIE), and ovarian endometrioma (OMA) (Bourdon *et al.*, 2024). While this surgical classification remains central to clinical practice, it is increasingly complemented by histopathological analysis and imaging techniques that provide additional insights into lesion characteristics and disease extent. One of the ongoing challenges in advancing endometriosis research and care is the development of physiologically relevant and reproducible model systems that adequately capture this phenotypic diversity. Established platforms, including non-human primate, rodent, and 2-dimensional *in vitro* models, have provided important insights into pathological processes. However, these models are often limited in their ability to capture the diversity of lesion subtypes, the complexity of the tissue microenvironment, and the variable hormonal, inflammatory, and fibrotic features which characterize endometriosis (Black *et al*., 2025).

Recent advances in 3-dimensional culture systems have led to the development of organoids, self-organizing tissue constructs derived from stem or progenitor cells which recapitulate the architecture and function of the tissue of origin (Marr *et al.*, 2025). Their ability to simulate complex cellular interactions makes organoids a powerful platform for advancing knowledge in developmental biology, regenerative medicine, and therapeutic innovation (Han *et al.*, 2024). As a complementary platform to existing *in vivo* and *in vitro* systems, these organoid models could ultimately support patient stratification and guide more effective, personalized interventions for managing pain and subfertility associated with endometriosis.

Organoid models derived from human endometrium have received focused research effort in recent years, and have contributed significantly to our understanding of endometrial physiology (Simintiras *et al.*, 2021; Fitzgerald *et al.*, 2023), hormone responsiveness (Garcia-Alonso *et al.*, 2021; Jiang *et al.*, 2024), pathology, (Brucker *et al.*, 2022; Zhang *et al.*, 2025b), and implantation biology (Shibata *et al.*, 2024; Yang *et al.*, 2025). This includes organoid models generated from eutopic endometrium from individuals with endometriosis, offering insights into disease-associated alterations in endometrial structure and function (Luddi *et al.*, 2020; Filby *et al.*, 2021; Liao *et al.*, 2024; Madasu *et al.*, 2024).

By contrast, organoid models derived from endometriosis lesions remain comparatively less developed, although a growing number of studies have begun to explore their feasibility and potential utility (Boretto *et al.*, 2019; Esfandiari *et al.*, 2021a,b; Mc Cormack *et al.*, 2021; Tan *et al.*, 2022; Zhang *et al.*, 2025a). While transcriptomic analyses of whole tissue suggest substantial similarity between patient-matched eutopic and ectopic tissues (Riaz *et al.*, 2024), the cellular composition, and architecture of lesions are markedly different. Lesions typically exhibit increased fibrosis and reduced proportions of epithelial cells, which are the primary population propagated in organoid systems. These features introduce distinct technical challenges for culture establishment, including differences in tissue dissociation, cell yield, and subsequent growth dynamics *in vitro*. Studies directly comparing organoids derived from matched eutopic and ectopic tissues have identified divergent transcriptional profiles, including altered expression of genes involved in extracellular matrix interaction, adhesion, and invasion (Boretto *et al.*, 2019). In addition, lesion-derived organoids from OMA show lower establishment efficiency and slower expansion compared to those derived from eutopic endometrium (Zhang *et al.*, 2025a). Together, these findings highlight the importance of developing models that reflect the biological complexity of endometriosis lesions.

However, despite growing interest in lesion-derived organoid models, no studies to date have examined how endometriosis phenotype influences organoid culture development nor clear criteria that define a ‘successful’ endometriosis organoid model. This study aimed to address these gaps by establishing and characterizing organoid cultures from a wide spectrum of endometriosis subtypes. It also investigated how factors such as biospecimen size, hormonal treatment status, and surgical phenotype influence organoid establishment and growth.

## Materials and methods

### Ethics approval

All biospecimens and clinical data were collected with Human Research Ethics Committee (HREC) approval from the South Eastern Sydney Local Health District (2021_ETH12472) and informed consent from participants.

### Patient recruitment and tissue collection

Eligible patients were those assigned female at birth over the age of 18 years who presented with symptoms or signs of endometriosis and were undergoing surgical treatment at Prince of Wales Private Hospital, Sydney. Exclusion criteria included pregnancy or suspected malignancy, and those with insufficient endometriotic tissue for donation. Tissue biospecimens for organoid development were only collected where there were sufficient additional lesions to allow for histopathological diagnosis and confirmation of endometriosis. Tissue biospecimens were surgically excised and placed in transport media containing Advanced DMEM/F12 (Gibco, No. 12634028, USA), 10% foetal bovine serum (FBS) (Scientifix, No. FBSFR-SOOJF, Australia), and 1% penicillin/streptomycin (Gibco, No. 15140122, USA). Biospecimens were placed on ice and transported to the laboratory within 4 h of collection. Biospecimens were cut into ∼5 mm^3^ and cryopreserved at −80°C in RPMI (Gibco, No. 11875093, USA) containing 40% FBS (Scientifix, No. FBSFR-SOOJF, Australia) and 10% DMSO (Sigma-Aldrich, No. D2650, USA).

### Clinical data collection

De-identified clinical data were extracted by an authorized research team member with access to medical records, including histological confirmation of endometriosis, primary presenting symptom, surgical history, revised American Society for Reproductive Medicine (rASRM) disease stage, patient age, and hormonal treatment status at the time of surgery.

### Tissue processing

Cryopreserved tissue vials were thawed and biospecimens weighed prior to processing according to process outlined in Boretto *et al.* (2019) and in accordance with the World Endometriosis Research Foundation Endometriosis Phenome and Biobanking Harmonisation Project for Experimental Models in Endometriosis Research (EPHect-EM-Organoids) (Marr *et al.*, 2025). Briefly, biospecimens were minced and rinsed in Ca^2+^/Mg^2+^-free PBS (Gibco, No. 14190144, USA) and transferred to a 50 ml tube containing Advanced DMEM/F12 (Gibco, No. 12634028, USA) supplemented with 20 µg/ml collagenase IV. The biospecimen was placed on a rocker at 180 rpm at 37°C, with mechanical titration conducted every 20 min with progressively smaller stripette tips (25 ml to 10 ml to 5 ml) with a minimum of 10 up-and-down passes per tip size. Digestion continued until no tissue fragments >1 mm^3^ were visible, for a maximum of 3 h (typical range 1–3 h). Progress was assessed visually. Digestion was stopped by doubling of media and large debris was removed with a 100 µm nylon filter (Corning, No. 352360), USA. The resulting suspension was centrifuged at 220*g* for 5 min at 4°C. The supernatant was removed, and the pellet resuspended in 70% growth-factor-reduced Matrigel (Corning, No. 356321, USA) according to cell density. Triplicate 20 µl Matrigel domes were plated in each well of a pre-warmed 24-well tissue-culture treated plate (Corning, No. 3524, USA) and returned to an incubator for Matrigel to set at 37°C for ∼15 min. Each well received 500 µl of prewarmed organoid medium (Supplementary Table S1) containing 1% antibiotic antimycotic (Sigma-Aldrich, No. A5955, USA).

### Organoid maintenance

Organoids were maintained in organoid media with a media change performed twice a week to every other day, dependent on cell growth. Upon reaching 80% organoid confluence, a mechanical passage was performed, wherein ice-cold 200 µl tips and media were used to disrupt Matrigel domes and break up organoids into small cell clumps. The resulting suspension was centrifuged at 200*g* for 5 min at 4°C to isolate cell pellets. Media was aspirated and pellets were resuspended in a 1:4 dilution of 70% Matrigel and re-plated in 20 µl domes as previously described. Each well received 500 µl of prewarmed organoid medium containing 10 µM Y-27632 (STEMCELL Technologies, No. 72304, Canada). For cryopreservation, organoid cultures at ≥80% confluence were harvested and Matrigel carefully removed to preserve 3-dimensional structure. Suspensions were centrifuged at 200*g* for 5 min at 4°C, and supernatant was removed. Pellets were resuspended in freezing medium containing 90% FBS and 10% DMSO at a density of ≥6 × 20 µl domes per cryovial. Organoids were transferred to cryovials and placed in a controlled-rate freezing container at −80°C. For revival, organoids were thawed and diluted dropwise into pre-warmed organoid medium prior to replating. An organoid model was only considered ‘successful’ when three criteria were met; (i) models formed 3-dimensional structures, (ii) they survived passage, and (iii) they could successfully be cryopreserved and thawed.

### Stromal cell separation and spheroid development

Any cells adherent to plastic wells were undisrupted during the organoid passage process. Where there were adherent cells retained on the plate surface after Matrigel was removed, this cell population was maintained in stromal cell media composed of DMEM/F12 (Gibco, No. 11320033, USA) containing 10% FBS with 1% GlutaMAX (Gibco, No. 35050061, USA) and 1% antibiotic/antimycotic (Sigma-Aldrich, No. A5955, USA). Cells were maintained until >80% confluence and passaged with 0.5% Trypsin-EDTA (Gibco, No. 15400054, USA) until confluence in a T25 flask (Corning, No. CLS430639, USA). Spheroids were created when required by plating 4×10^3^ cells per well in a round-bottom ultra-low attachment plate (Corning, No. 7007, USA) and allowed to form for 4 days with no media change.

### Immunohistochemistry and immunofluorescence

Organoids or spheroids were collected after defined time periods and centrifuged at 200*g* for 5 min to form pellets. Media was aspirated and models were resuspended in warmed HistoGel (Epredia, No. 22-110-678, USA). HistoGel domes were allowed to set at room temperature and fixed in 4% formalin for 1 h before being transferred to 70% ethanol. Domes were embedded in paraffin and sectioned for haematoxylin and eosin (H&E) staining.

Immunohistochemistry (IHC) and immunofluorescence (IF) were conducted on the Leica Bond platform following heat-induced epitope retrieval. IHC was conducted for oestrogen receptor alpha (ERα) (1:100, GeneTex GTX29269, USA), progesterone receptor (PR) (1:1000, Cell Signalling Technology No. 8757, USA), and Ki67 (1:3000, Cell Signalling Technology, No. 9449, USA). IF was conducted with rabbit CD10 (1:500, Abcam, ab256495, UK), mouse E-cadherin (1:500, Abcam, ab76055, UK), counterstained with DAPI (Sigma-Aldrich, D9542, USA), 555-conjugated donkey anti-rabbit IgG (1:1000, Invitrogen, A-21572, USA), and 488-conjugated donkey anti-mouse IgG (1:1000, Invitrogen, A-210C2, USA).

### Microscopy

All slides were scanned at 40× objective brightfield or fluorescence on an Olympus VS200 slide scanner. Ki67 positivity was calculated by positive cell detection in QuPath (version 0.4.4), using the following settings: optical density (OD) sum; requested pixel size 0.5 µm; background radius 0.8 µm; median filter radius 0 µm; sigma 1.5 µm; min/max area 10/400 µm; threshold 0.05; max background intensity 2.0; cell expansion 5 µm; and threshold value for positivity set to 0.05 DAB OD mean for all slides.

### Shape analysis

The H&E staining images were analysed using a custom algorithm written in Python 3.9 to extract relevant organoid shape features, including circularity, normalized perimeter, and normalized entropy. Raw TIFF images were manually cropped to ensure each image contained only one organoid. At this stage, images were also categorized according to the organoid’s overall morphology. ‘Cystic’ organoids were defined by a well-organized, structured border, and the presence of an internal lumen. Structures lacking defined organization were classified as ‘solid’, while organoids exhibiting both structured and unstructured regions were categorized as ‘mixed’.

Image segmentation was performed to identify pixels corresponding to each organoid. The segmentation approach varied depending on the qualities of the image and organoid morphology, as summarized in Supplementary Table S2. In most cases, global thresholding was applied, with segmentation algorithms selected based on organoid type. Global image thresholding was performed on cystic organoids using Otsu’s method (Otsu, 1979), which determines the optimal threshold by minimizing the intra-class variance of pixel intensities. This was implemented using the threshold_otsu function from the Python scikit-image library (version 0.16.2, Sydney, Australia). For non-cystic organoids, mean thresholding was applied. In cases where standard methods failed to yield accurate segmentation, alternative approaches were selected empirically (see Supplementary Table S2 for details).

Following segmentation, shape metrics were computed. Circularity (Equation 1) quantified how round an organoid was, with 1 corresponding to a perfect circle. In Equation 1, area was defined as the total number of pixels classified as being part of the organoid, and the perimeter represented the length of the external border. Perimeter measurements were obtained using the scikit-image Python package, employing a 4-pixel neighbourhood.


(1) $$\mathrm{Circularity}=4\pi \cdot (\frac{\mathrm{Area}}{{\mathrm{Perimeter}}^{2}})$$

The perimeter of the organoid employed as a metric to quantify shape irregularity. To account for size-dependent variability, the perimeter was normalized by the area of the organoid. Entropy was used as a measure of morphological complexity, reflecting the heterogeneity in the distribution of grey levels within the image. The scikit-image Python package was used to calculate the entropy image, where each pixel value corresponds to the entropy within a local neighbourhood defined by a disk of radius 5 pixels. The normalized entropy for each image was then calculated as the proportion of pixels with entropy values greater than zero relative to the total image size.

### Statistical analysis

The statistical difference between success and failure of culture conditions by clinical parameters was calculated using Pearson’s chi-square in SPSS (version 30.0.0, Sydney, Australia). Where >20% of cells had an expected count of <5, Fisher’s exact test was applied. Age was analysed categorically using two groups based on the cohort’s median age.

The relationship between organoid culture success and biospecimen mass was assessed using a linear mixed model in R studio (version 4.5.0, Sydney, Australia), with establishment as a fixed effect and patient as a random effect to account for multiple measurements per patient. Inference was performed using the lme4 package (version 1.1.37), and pairwise comparisons were conducted using emmeans. Differences in organoid morphology among phenotypes were analysed using linear mixed models in R, with phenotype as a fixed effect and patient as a random effect. Inference was performed as above using parameters and emmeans.

All statistical analyses were conducted in R unless otherwise specified. Data visualization was performed in GraphPad Prism (version 9.3.1, Sydney, Australia). A *P*-value <0.05 was considered statistically significant.

## Results

### Endometriosis organoid cultures can be established from diverse clinical presentations of disease

**Figure 1.**
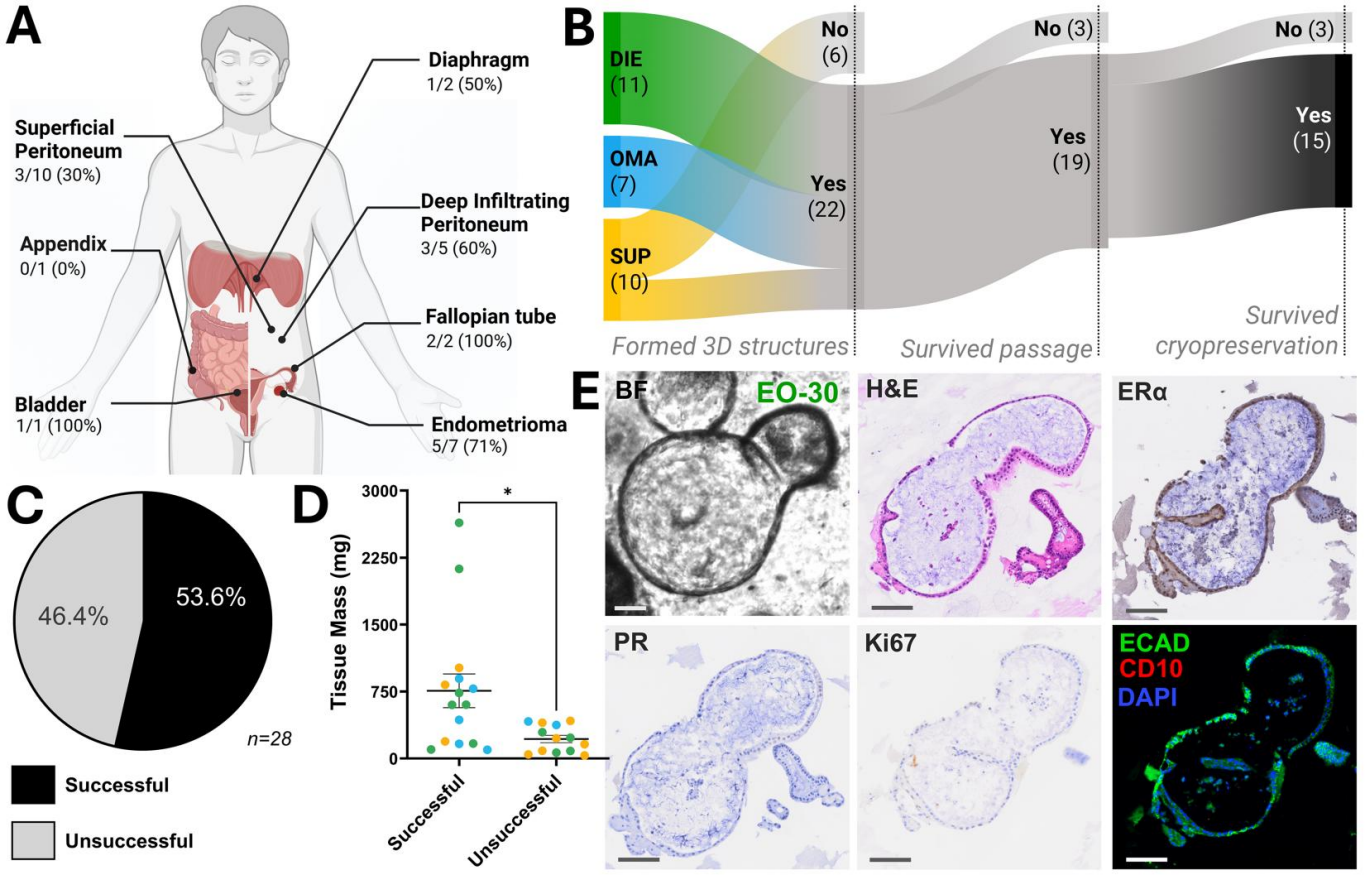
**Overview of organoid generation from endometriosis biospecimens highlighting workflow, success criteria, and characterization.** (**A**) Organoid models were successfully established from endometriosis biospecimens from multiple anatomical sites, including the peritoneum, bladder, diaphragm, fallopian tube, and ovary (endometriomas). (**B**) Sankey diagram illustrating the tissue processing pipeline for organoid establishment. Input biospecimens are grouped by surgical disease phenotype: deep infiltrating endometriosis (DIE, green), ovarian endometrioma (OMA, blue), and superficial peritoneal endometriosis (SUP, yellow). The diagram tracks sample progression through three successive binary success criteria: formation of 3-dimensional structures, survival following passaging, and survival following cryopreservation. (**C**) Overall organoid establishment success rate exceeded 50%. (**D**) Biospecimen mass was significantly associated with successful organoid generation (*P*≤0.05). (**E**) Representative images of organoid model EO-30 showing morphology and biomarker expression (left to right, top to bottom): brightfield microscopy, haematoxylin and eosin (H&E) staining, oestrogen receptor alpha (ERα), progesterone receptor (PR), Ki67, E-cadherin, CD10, and DAPI nuclear staining. Scale bar represents 100 µm. Asterisks (*) indicates *P* < 0.05. Figure partially created in BioRender. Abbott, J. (2025) https://BioRender.com/hcv5lhc.

A total of 28 distinct endometriosis biospecimens were collected from 23 participants (Fig. 1A, Table 1). Given the *a priori* success criteria, 22/28 (78.6%) lesions formed 3-dimensional structures, of which 19/22 (86.4%) were successfully passaged (Fig. 1B). Of these, 15 organoid models remained viable after cryopreservation (78.9%). This corresponds to an overall organoid establishment rate of 53.57% (15/28 lesions, Fig. 1C). Higher biospecimen mass was significantly associated with successful organoid establishment overall (Fig. 1D, *P* = 0.012), with similar trends across phenotypes (Supplementary Fig. S1). The smallest tissue biospecimen yielding a successful model weighed 98 mg (Table 1, Supplementary Fig. S1). One organoid model (EO-28) failed embedding and has no representative microscopy images due to technical issues.

**Table 1.** Lesion characteristics associated with organoid establishment.

Sample ID	Phenotype	Size (mg)	Formed 3-dimensional structures	Survived passage	Survived cryopreservation	Successful model
EO-12.1	SUP	45	No	N/A	N/A	No
EO-12.2	DIE	85	Yes	Yes	No	No
EO-14	DIE	66	Yes	No	N/A	No
EO-16	OMA	433	Yes	Yes	Yes	Yes
EO-17	DIE	101	Yes	Yes	Yes	Yes
EO-20	SUP	226	No	N/A	N/A	No
EO-22	DIE	735	Yes	Yes	Yes	Yes
EO-26	SUP	191	Yes	Yes	Yes	Yes
EO-27	SUP	87	No	N/A	N/A	No
EO-28	DIE	603	Yes	Yes	Yes	Yes
EO-30	DIE	601	Yes	Yes	Yes	Yes
EO-31	OMA	376	Yes	Yes	No	No
EO-32	DIE	2124	Yes	Yes	Yes	Yes
EO-35	SUP	34	No	N/A	N/A	No
EO-36	SUP	825	Yes	Yes	Yes	Yes
EO-39.1	SUP	401	Yes	No	N/A	No
EO-39.2	DIE	167	Yes	Yes	Yes	Yes
EO-42	DIE	232	Yes	Yes	No	No
EO-45.1	SUP	422	No	N/A	N/A	No
EO-45.2	DIE	295	Yes	Yes	No	No
EO-47.1	OMA	782	Yes	Yes	Yes	Yes
EO-47.2	DIE	2640	Yes	Yes	Yes	Yes
EO-48	SUP	1016	Yes	Yes	Yes	Yes
EO-51	OMA	165	Yes	Yes	Yes	Yes
EO-61	OMA	416	Yes	No	N/A	No
EO-64	SUP	159	No	N/A	N/A	No
EO-66.1	OMA	98	Yes	Yes	Yes	Yes
EO-66.2	OMA	895	Yes	Yes	Yes	Yes

EO, endometriosis organoid; SUP, superficial peritoneal endometriosis; DIE, deep infiltrating endometriosis; OMA, endometrioma; N/A, not applicable.

**Figure 2.**
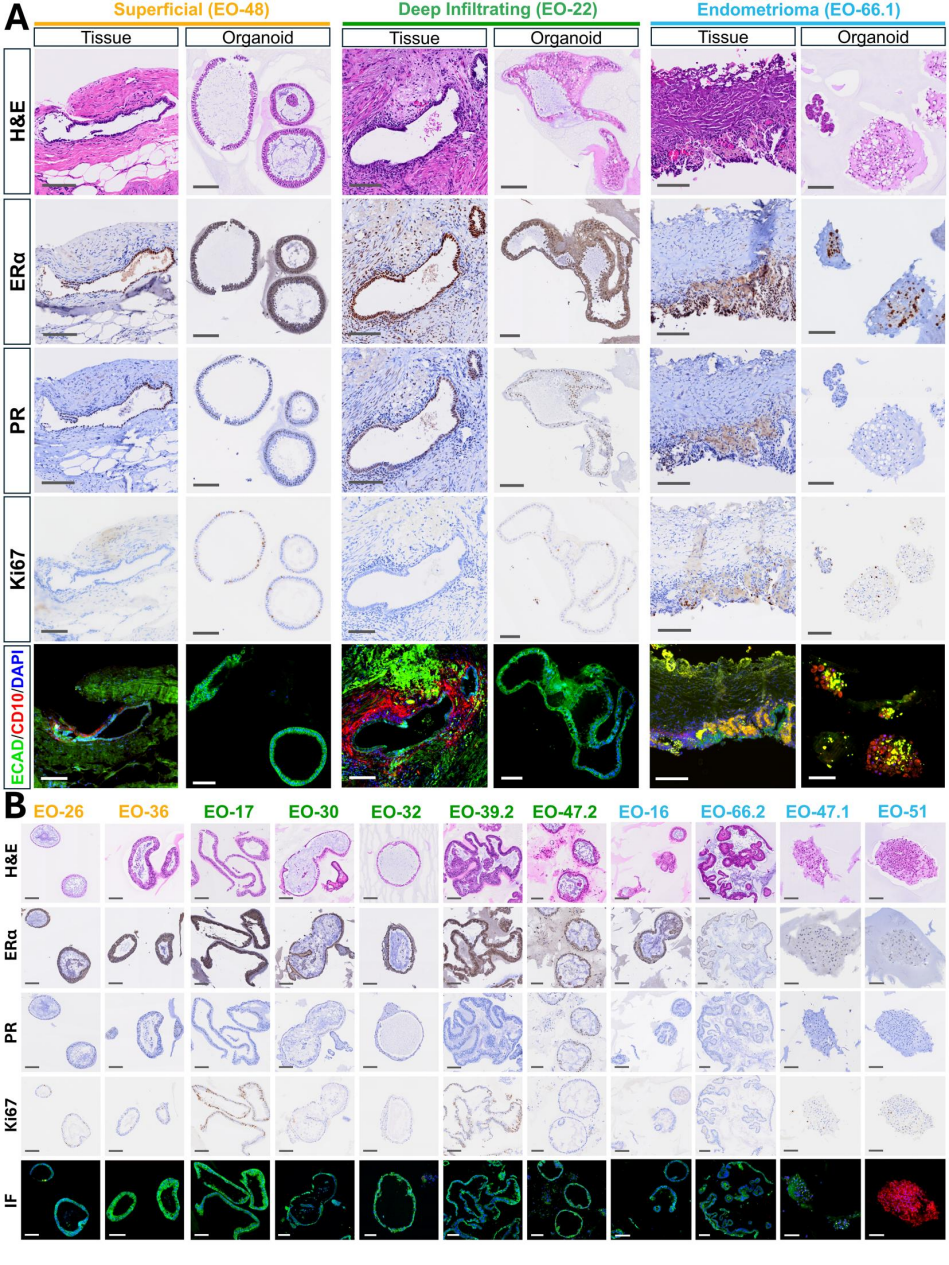
**Endometriosis organoids recapitulate the primary tissues they are derived from.** (**A**) Representative images from one patient per surgical phenotype (superficial peritoneal [SUP], deep infiltrating endometriosis [DIE], ovarian endometrioma [OMA]) comparing primary tissue and matched organoid models. Panels show haematoxylin and eosin (H&E) staining, oestrogen receptor alpha (ERα), progesterone receptor (PR), Ki67 proliferation marker, and immunofluorescence for E-cadherin (epithelial marker) and CD10 (stromal marker). Organoids preserve key histological features and marker expression patterns of the originating tissues. (**B**) Immunohistochemical and immunofluorescence staining of organoid models from multiple patients across phenotypes for H&E, ERα, PR, Ki67, E-cadherin, and CD10, demonstrating morphological and molecular heterogeneity within organoid cultures. Sample IDs are coloured by phenotype: SUP=yellow, DIE=green, OMA=blue. Scale bar represents 100 µm.

Endometriosis organoids faithfully recapitulated the primary tissues they were derived from, in both histological features (Fig. 2A, Supplementary Fig. S2) and expression of ERα, PR, Ki67, E-cadherin, and CD10 (Fig. 2B).

When stratified by clinical characteristics, organoid establishment success was not significantly associated with disease stage, location, recurrence status, patient age, or primary symptomatology (Table 2). However, patients undergoing hormonal treatment at the time of tissue collection were significantly less likely to yield a successful organoid model than those not on hormonal treatment (Table 2, *P* = 0.003).

**Table 2.** Association between organoid model success and patient clinical characteristics.

	Number successful, (%)	Number unsuccessful, (%)	*P*-value
**rASRM stage**			
Early (I/II)	6 (42.9)	8 (57.1)	0.256
Late (III/IV)	9 (64.3)	5 (35.7)	
**Disease location**			
Pelvic peritoneum	6 (40.0)	9 (60.0)	0.409
Endometrioma	5 (71.4)	2 (28.6)	
Extrapelvic	4 (66.7)	2 (33.3)	
**Surgical history**			
Primary	4 (57.1)	3 (42.9)	1.000
Recurrent	11 (52.4)	10 (47.6)	
**Hormone treatment**			
Yes	3 (23.1)	10 (76.9)	**0.003**
No	12 (80.0)	3 (20.0)	
**Age** *			
≤37	7 (46.7)	8 (53.3)	0.431
>37	8 (61.5)	5 (38.5)	
**Primary symptom**			
Pain	11 (47.8)	12 (52.2)	0.333
Non-pain	4 (80.0)	1 (20.0)	

rASRM, revised American Society for Reproductive Medicine, non-pain category includes subfertility and abnormal uterine bleeding.

Statistical significance indicated in bold.

* Age grouped based on cohort median (37 years).

### Cell populations will self-organize and purify over time

**Figure 3.**
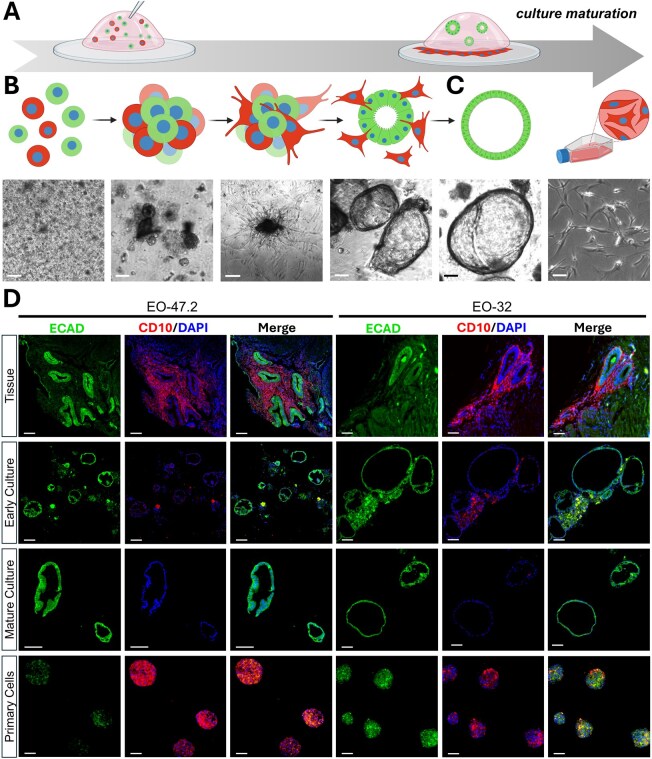
**Purification of epithelial cell content in organoid models over time in culture.** (**A**) A heterogeneous mix of tissue-derived epithelial (green) and stromal (red) cells is embedded in Matrigel, with phenotypic separation observed during culture maturation. (**B**) Representative brightfield images of EO-39.2 across culture progression: from initial plating through late P0, P1, and P2. Cells transition from isolated or small clusters to structured organoids with emerging spindle-shaped protrusions. These spindle-shaped cells invade the Matrigel and adhere to the base of the culture plate. (**C**) At passage 3 (P3), cultures show a homogenous organoid population and a distinct 2-dimensional layer of spindle-shaped cells. (**D**) Microscopy images of EO-47.2 and EO-32 showing the primary tissue, early culture, mature culture, and spheroids created from primary 2-dimensional cells. Tissue is composed of E-cadherin-positive (green) endometrium-like epithelium admixed with CD10-positive (red) stromal cells. Early passage cultures show the development of E-cadherin-positive organoid structures with peripheral CD10-positive cell clumps. Over consecutive passage to a mature culture, only the E-cadherin-positive organoid structures remain. Primary cells which migrated through the Matrigel and generated a 2-dimensional culture were collected and used to create CD10-positive spheroids. Scale bar represents 100 µm. Figure partially created in BioRender. Abbott, J. (2025) https://BioRender.com/hcv5lhc.

Cell suspensions from primary tissues were cellularly heterogeneous, but cellular populations self-selected over time (Fig. 3A). At the first few days of culture, cells began to cluster in Matrigel, with elongated and spindle-like protrusions developing from the clusters within the first 2 weeks of culture (Fig. 3B). As the culture matured, spindle-shaped cells emerged from rounded organoids, and migrated through the Matrigel to attach to the bottom of the dish (Fig. 3C). Resulting cultures contained organoids within the Matrigel domes, and 2-dimensional primary cells adherent to the plate which could be separated and cultured separately (Fig. 3C). IF staining confirmed that spheroids created with 2-dimensional primary cells were CD10+ (Fig. 3D).

### Organoids can be derived from all phenotypes of endometriosis, but show variable success and morphology

**Figure 4.**
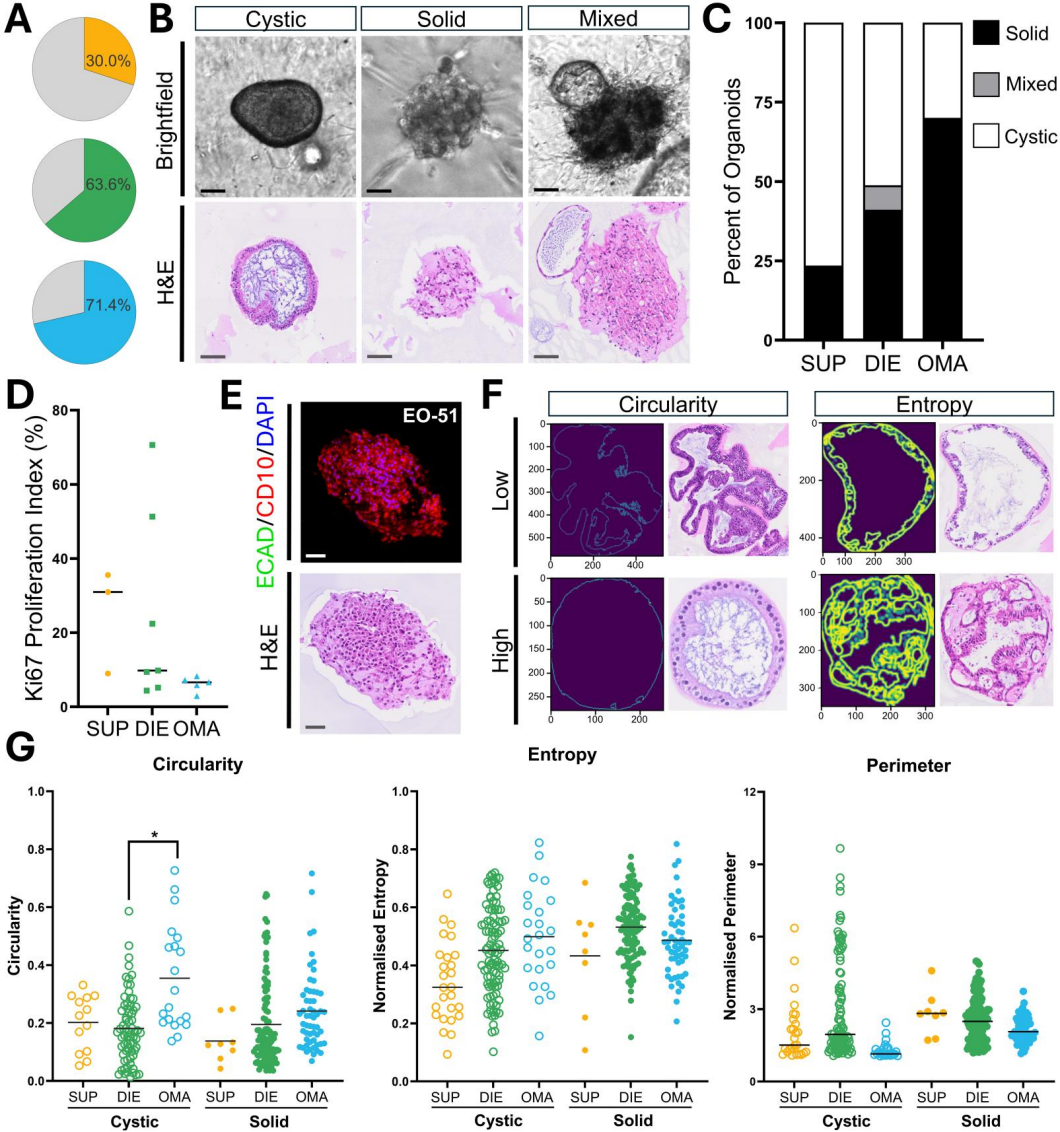
**Organoid success rates and morphological diversity across endometriosis surgical phenotypes.** (**A**) Organoid establishment success rates stratified by surgical phenotype: superficial peritoneal (SUP, yellow), deep infiltrating endometriosis (DIE, green), and ovarian endometrioma (OMA, blue). (**B**) Organoid morphologies are classified as cystic, solid, or mixed. (**C**) Distribution of organoid morphology by surgical phenotype. (**D**) Ki67 proliferation index of organoids by surgical phenotype. (**E**) Representative case (EO-51) showing organoids composed of CD10-positive stromal cell organoids. (**F**) Example illustrating the assessment of organoid circularity and entropy. (**G**) Quantitative comparison of organoid circularity, entropy, and perimeter across surgical phenotype and morphological subtypes. Each dot represents a single organoid. Scale bar represents 100 µm. *P ≤ 0.05.

When stratified by surgical phenotype, SUP lesions exhibited the lowest rate of organoid establishment, with successful models derived from only 3 of 10 biospecimens (30%, Fig. 4A). By contrast, higher success rates were observed for OMA (5/7, 71.4%) and DIE (7/11, 63.6%) (Fig. 4A). When only accounting for DIE in extrapelvic locations, success rates were similar (4/6, 66.7%). The resulting organoids demonstrated three distinct morphological categories: cystic structures with a defined lumen, solid structure lacking a lumen, and mixed morphology exhibiting features of both (Fig. 4B, Supplementary Fig. S3). Organoids from SUP lesions were predominantly cystic in appearance, while DIE organoids were the only group to exhibit a mixed morphology (Fig. 4C). OMA-derived organoids were predominantly solid structures (Fig. 4C) and were the only group to generate organoids containing stromal cell populations (Fig. 4E). Quantitative morphological analysis of cystic organoids revealed cystic OMA organoids were significantly more circular (*P* = 0.045) compared to those derived from DIE biospecimens (Fig. 4G).

### Organoids can be successfully generated from patients receiving hormonal treatment, but are sensitive to cryopreservation

**Figure 5.**
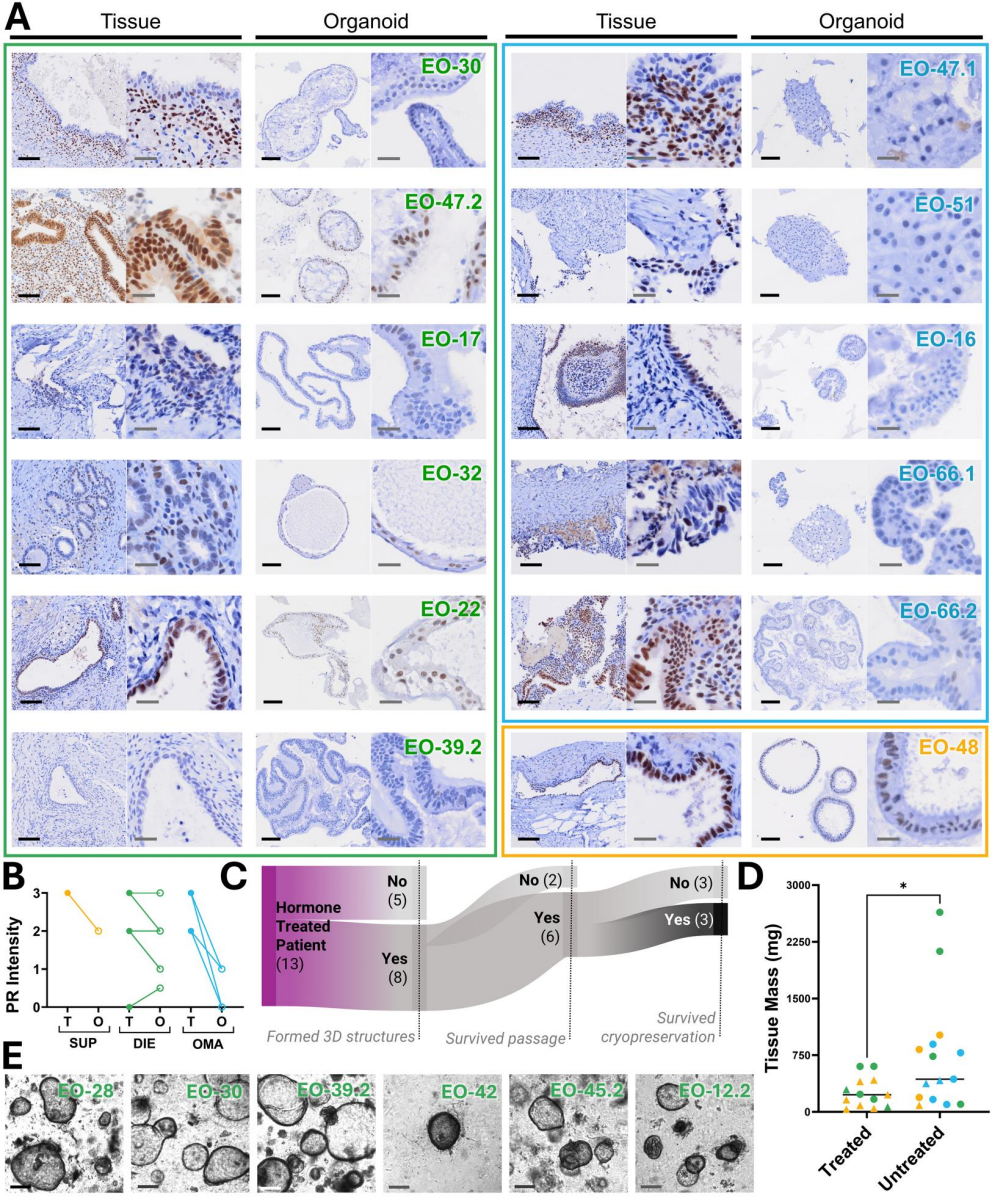
**Progesterone receptor expression and organoid establishment across endometriosis subtypes in hormone-treated patients.** (**A**) Representative immunohistochemistry images of progesterone receptor (PR) expression in primary tissues and organoid models. EO-26, EO-36 not represented duet to no endometriosis in primary tissue blocks at the level analysed. Black scale bar: 100 µm Grey scale bar: 20 µm. (**B**) PR staining intensity in primary endometriosis tissue versus organoid models. Superficial (SUP, yellow) and deep infiltrating endometriosis (DIE, green) organoids show equal or slightly reduced PR expression than primary tissues. Endometrioma (OMA, blue) organoids show marked reduction in PR expression versus primary tissue. (**C**) Summary of organoid generation workflow for hormone-treated cases, indicating success rates at each stage of the pipeline. (**D**) Lesions mass was significantly lower in hormone-treated patients compared to untreated patients. Colour indicates surgical phenotype: superficial peritoneal (SUP, yellow), deep infiltrating (DIE, green), and endometrioma (OMA, blue). Line represents medians. *P≤0.05. (**E**) Brightfield images of cultures from hormone-treated patients which successfully formed 3-dimensional structures and survived passage. Scale bar represents 250 µm.

Nine out of twenty-one (42.9%) patients were receiving hormonal treatment at the time of surgery, representing 13 biospecimens (6 SUP, 7 DIE). Biospecimens from patients receiving hormonal treatment were significantly smaller in mass compared to those from untreated patients (*P *= 0.0378, Fig. 5D). Three-dimensional structures successfully formed in 6 of these 13 biospecimens, (46.2%), with a higher success rate observed in DIE lesions (5/7) than SUP (1/6) (Fig. 5C). In all cases, where matched SUP and DIE biospecimens were collected from the same patient, only the DIE lesions formed 3-dimensional structures. Upon cryopreservation, only half of the organoids remained viable upon thawing (3/6, 50%) (Fig. 5C).

Across organoid models, PR expression was generally well retained in SUP and DIE-derived organoids, with 7/7 (100%) showing no change or only a slight reduction compared to matched primary tissue (Fig. 5A and B). OMA-derived organoids exhibited larger decreases in PR expression, with 4/5 cases showing reductions of two or more points on a four-point intensity scale (Fig. 5A and B).

## Discussion

This study is the first to systematically evaluate the feasibility of generating organoid models across a broad spectrum of surgically defined endometriosis phenotypes, including the first descriptions of organoids from less commonly sampled sites including the diaphragm, bladder, and fallopian tube.

Using a single, previously validated culture method and medium (Boretto *et al.*, 2019), notable differences were observed in organoid establishment efficiency among surgical phenotypes. Organoid establishment was less successful from SUP biospecimens compared to DIE and OMA, suggesting inherent differences in cellular viability or tissue composition. SUP biospecimens were less likely to form 3-dimensional structures, failing in 60% of cases (6/10, Fig. 1B). Although no significant difference in tissue mass was observed between phenotypes, within the SUP group, successful organoid cultures were associated with significantly greater tissue mass than unsuccessful attempts (Supplementary Fig. S1). This may reflect lower endometriotic cell purity in SUP lesions, where smaller specimens contain proportionally fewer viable epithelial or stromal cells of endometriotic origin, an observation is consistent with cancer organoid studies, where tumour purity critically influences organoid establishment efficiency (Dijkstra *et al.*, 2020).

To enable batching of biospecimen processing and provide a robust, practical workflow aligned with common biobanking practices, tissue samples in this study were cryopreserved prior to processing. This approach enhances the feasibility and accessibility of organoid models for researchers, particularly those reliant on third-party tissue sources. Whether cryopreservation influenced organoid establishment rates in our cohort remains speculative. Transparent reporting of establishment success in endometriosis organoids is limited, with one study reporting a 75% success rate (18/24) from OMA samples, consistent with our findings (Zhang *et al.*, 2025a). Comparable data for other phenotypes are lacking; however, it is plausible that the lower establishment rate observed in superficial endometriosis reflects greater sensitivity to cryopreservation-related stress, as reported in other organoid systems.

Additionally, organoids derived from OMA were smaller in size and had a lower proliferative capacity compared to those from DIE and SUP, as indicated by reduced Ki67 expression. These findings underscore the necessity for subtype-specific culture conditions for endometriosis organoids, consistent with *ex vivo* data revealing distinct differentiation signalling pathways in peritoneal (SUP, DIE) versus OMA lesions (Tan *et al.*, 2022). This aligns with broader evidence that organoids from different female reproductive tissues rely on divergent growth factor milieus: ovarian and fallopian tube organoids typically depend on Wnt3a (Kessler *et al.*, 2015; Kopper *et al.*, 2019), whereas endometrium organoids do not (Boretto *et al.*, 2017; Turco *et al.*, 2017). The low-Wnt media used in this study was based on the concept of endometriosis is a ‘endometrial disease’ (Boretto *et al.*, 2019), yet recent findings indicate that OMA organoids require alternative conditions, such as Wnt3a-enriched and FGF-reduced media (Zhang *et al.*, 2025a). Collectively, these data highlight that future efforts should prioritize developing culture protocols that reflect the distinct molecular and cellular characteristics inherent to each disease subtype to improve physiological relevance and reproducibility.

Unlike previous investigations, this cohort was not restricted by hormonal treatment status or menstrual phase, thereby more accurately reflecting the clinical heterogeneity of endometriosis patients. Many studies limit tissue sampling to the proliferative phase (Esfandiari *et al.*, 2021a,b), despite the known asynchrony between eutopic endometrium and ectopic lesions across the menstrual cycle (Colgrave *et al.*, 2020). To better mirror the clinical population undergoing surgery, future tissue collection should include biospecimens from all menstrual phases. Furthermore, since hormonal therapy is a recommended first-line treatment for patients with endometriosis worldwide (Kalaitzopoulos *et al.*, 2021), restricting tissue acquisition to untreated cases may compromise the external validity of these models. This would disproportionately affect specific patient populations, with almost 90% of trans men, 70% of adolescents and deeply invasive rectosigmoid endometriosis cases, and about half of those undergoing hysterectomy receiving hormonal treatment prior to endometriosis surgery (Ferrando *et al.*, 2021; Casarin *et al.*, 2023; Arena *et al.*, 2024; Bergus *et al.*, 2025). Consequently, patients who may have more complex or unique healthcare needs could be underrepresented or excluded from research models, limiting the applicability of findings to these important subgroups. Although hormonal treatment was associated with significantly smaller biospecimen size and reduced likelihood of model success, these findings demonstrate that it is still possible to successfully generate organoid models from patients on hormonal agents. Modelling the full spectrum of disease presentations, including treatment-exposed patients, is essential to enhance the translational relevance of these pre-clinical systems.

Endometriosis organoids demonstrated considerable morphological heterogeneity across all subtypes, with variation in circularity, entropy, and size-related metrics. Many organoids exhibited low circularity and increased shape irregularity, especially those with higher entropy and larger perimeters. While cystic organoids derived from DIE appeared more structurally disordered than those from SUP, this trend did not reach statistical significance, potentially due to limited sample size in the SUP group. Notably, previous research has shown OMA organoids to be more morphologically disordered than patient-matched eutopic endometrium organoids (Zhang *et al.*, 2025a), while in this study, DIE organoids showed even greater irregularity than OMA. This gradient of morphological complexity may reflect the invasive and disorganized cellular behaviour characteristic of DIE lesions *in vivo*, contrasting with the more encapsulated nature of OMAs.

This morphological diversity is not only indicative of cellular behaviour *in vitro* but also aligns with findings from other fields, where organoid morphology has proven to be a valuable functional readout. For example, lung cancer bone metastasis organoids with distinct morphologies have been correlated with EGFR mutation status and resistance to tyrosine kinase inhibitors (Hu *et al.*, 2024). Similarly, in oral cancer organoids, compact or ‘grape-like’ morphologies have been associated with specific transcriptional programs, recurrent mutations in *TP53*, *NOTCH1*, and *FAT1*, and poorer patient survival (Lee *et al.*, 2025). These studies underscore the value of morphological profiling as a means to connect cellular phenotype with genetic drivers and clinical outcomes. Extending this concept to endometriosis, the present findings reveal significant morphological heterogeneity within organoids that likely reflects critical aspects of disease biology, including invasive potential and cellular organization. As the first analysis of organoid morphology in endometriosis, this study provides a foundation for future research aimed at integrating morphological characteristics with genetic and clinical data to deepen understanding of endometriosis pathophysiology.

However, it should be noted that these morphometric analyses were based on 2-dimensional microscopy images of a single plane, a methodology which is intrinsically limited in capturing the resolution required to understand the complex architecture of a 3-dimensional structure. Future studies should utilize advanced imaging modalities and AI-driven segmentation tools capable of live-cell, 3-dimensional analysis. Emerging platforms such as OrganoID and LabelFreeTracker leverage deep learning to dynamically quantify organoid morphology and behaviour in 3-dimensional, offering more faithful representations of structural heterogeneity and luminal formation over time (Matthews *et al.*, 2022; Kok *et al.*, 2025). When integrated with clinical data and patient-reported outcomes, such as fertility history and pain signatures, these morphological signatures could offer a functional link between *in vitro* phenotype and *in vivo* disease behaviour. In doing so, endometriosis organoids could emulate the translational potential seen in cancer organoid systems, serving as platforms for patient stratification, biomarker discovery, and therapeutic prediction.

This study has several limitations. Histological confirmation of input material is a challenge in organoid research and may be particularly difficult in endometriosis due to its multifocal nature and the small, heterogeneous appearance of lesions. Even macroscopically visible lesions may lack classical endometrial-type glands or stroma, especially when fibrotic or atypical. A recent study that examined whole lesions using both superficial and deep sectioning reported a 20% rate of histologically negative lesions (Chen *et al.*, 2025). To mitigate this, multiple lesions of the same phenotype were pooled in 12 of 28 cases, increasing the likelihood of capturing endometriosis tissue. In cases where only a single lesion was available, 9 of 16 samples exceeded 200 mg, improving confidence in tissue adequacy despite the absence of pooling. Direct cell-type quantification was not feasible due to limited tissue volume; however, adjacent lesions collected for routine clinical pathology were used to confirm the presence of endometriosis. This approach reflects the most practical and clinically aligned strategy for tissue verification under current constraints. Nonetheless, pooling lesions from the same patient may obscure intrapatient heterogeneity in hormone receptor expression (Colgrave *et al.*, 2024) or driver mutations (Praetorius *et al.*, 2022). It remains unclear how this could influence the biological fidelity of these models and their applicability in precision medicine applications.

Further, the culture conditions used to develop endometriosis organoid models selectively enrich for epithelial cells. While this enables detailed investigation of epithelial biology, a primary cell population currently underrepresented in endometriosis research (Gunther *et al.*, 2025), it also introduces a key limitation. In endometriosis lesions, coordinated interactions between epithelial, stromal, and immune cells underpin key aspects of lesion biology, including immune crosstalk and inflammatory signalling (Burns *et al.*, 2025). The absence of these interacting cell types in monoculture organoid systems limits the extent to which such models can recapitulate the cellular complexity and microenvironmental dynamics of *in vivo* lesions. Current endometriosis organoids lack stromal, immune, vascular, and neural components, and should therefore be viewed as complementary rather than comprehensive tools. Nonetheless, they offer a valuable foundation for the development of more physiologically relevant *in vitro* systems, including co-cultures and assembloids that may better model multicellular interactions.

Emerging work in other inflammatory and reproductive conditions provides a roadmap for expanding the biological fidelity of endometriosis organoid systems. For example, epithelial-lymphocyte co-cultures have been used to model immune interactions in Crohn’s disease (Hammoudi *et al.*, 2022). Recent endometrial tissue engineering platforms offer valuable techniques for incorporating stromal cells into organoid cultures (Gnecco *et al.*, 2020). Adapting these approaches to endometriosis models could enable systematic investigation of lymphocyte-epithelial and stromal-epithelial interactions, such as the effects of varying stromal-to-epithelial cell ratios or incorporating macrophages with distinct activation states to better recapitulate diverse lesion microenvironments. These stromal cell lines could be derived directly from the same lesion, as demonstrated in this study by the incidental outgrowth of stromal cells that migrated through the Matrigel. While the exact conditions supporting this stromal outgrowth remain unclear, factors such as lesion size, anatomical subtype, recurrence status, and initial cellular composition may all play a role. Moreover, the Matrigel-based collection method may bias selection towards more motile or invasive stromal subpopulations. Although this could skew the culture towards aggressive phenotypes, it may also offer a valuable platform to interrogate functional attributes associated with lesion progression *in vitro*. Future studies incorporating eutopic endometrium-derived material alongside lesion-derived organoids will be essential to deepen comparative analyses, particularly with respect to differences in cellular aggressiveness, invasive capacity, hormone responsiveness, and treatment resistance. Such comparative work will be critical to delineate lesion-specific phenotypes from eutopic endometrial biology, and to better understand mechanisms underpinning disease establishment and progression.

In conclusion, this work demonstrates that organoid models can be successfully developed from a broad spectrum of endometriosis subtypes, but that their success is influenced by biospecimen size, anatomical location, hormonal exposure, and disease phenotype. These models represent a promising platform to explore the cellular and molecular diversity of endometriosis and offer opportunities to investigate subtype-specific biology *in vitro*. By refining culture conditions and linking organoid characteristics with clinical data such as hormonal treatment, lesion type, pain scores, and fertility outcomes, endometriosis organoids may support future efforts in patient stratification and therapeutic development. Ultimately, by modelling the intrinsic heterogeneity of endometriosis, organoids may inform the development of personalized therapeutic strategies and improve future patient care.

## Supplementary Material

deaf230_Supplementary_Data_File_S1

deaf230_Supplementary_Figure_S1

deaf230_Supplementary_Figure_S2

deaf230_Supplementary_Figure_S3

deaf230_Supplementary_Table_S1

deaf230_Supplementary_Table_S2

## Data Availability

The data underlying this article include clinically annotated patient information and cannot be shared publicly to protect participant privacy. De-identified annotation and representative summary data are available in the Supplementary Data File S1, and additional quantitative and image-derived data will be shared on reasonable request to the corresponding author.
