# Supplementary material for: Patient-derived epithelial cell organoids mimic the phenotypic complexity of endometriosis subtypes
Source: Hum Reprod. 2025 Nov 27;41(2):262–74. doi: 10.1093/humrep/deaf230 (PMC12864149; doi:10.1093/humrep/deaf230)
Supplement: deaf230_Supplementary_Figure_S1 [file deaf230_supplementary_figure_s1.pdf]

Human Reproduction, 2025, 1–1
https://doi.org/10.1093/humrep/deaf230
Supplementary Data

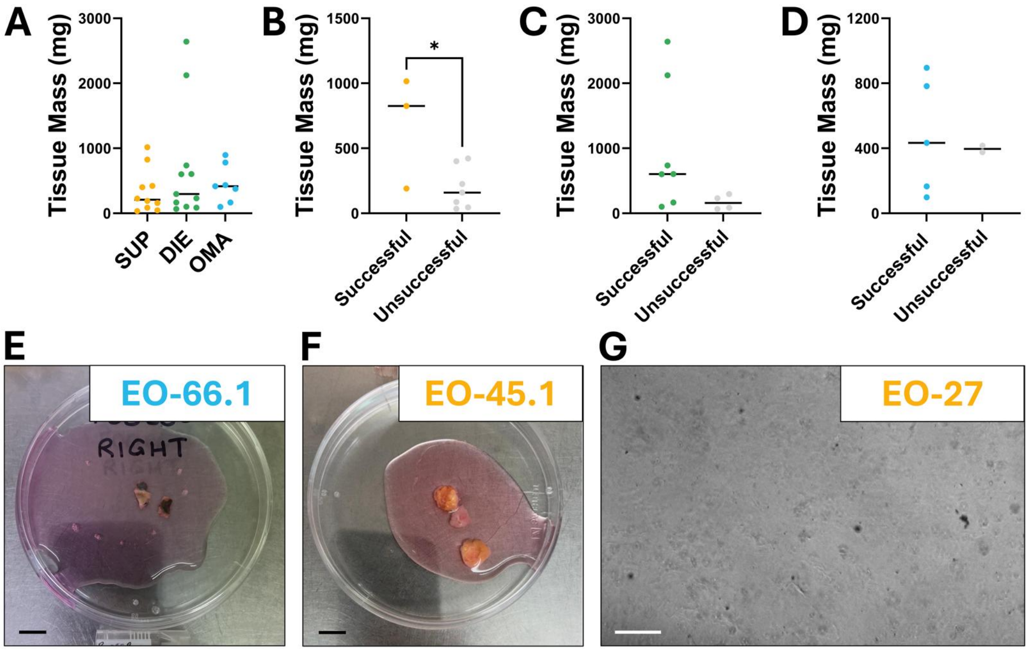

Supplementary Figure S1. Tissue mass influences organoid establishment. (**A**) Biospecimen mass by phenotype. SUP, superficial endometriosis; DIE, deep infiltrating endometriosis; OMA, endometrioma. (**B**) Superficial biospecimen mass by organoid establishment success. $P<0.05$. (**C**) Deep infiltrating endometriosis biospecimen mass by organoid establishment success. (**D**) Endometrioma biospecimen mass by organoid establishment success. (**E**) Smallest biospecimen which successfully established an organoid model, EO-66.1. Scale bar=1 cm. (**F**) Largest biospecimen which was unsuccessful in establishing an organoid model, EO-45.1. Scale bar=1 cm. (**G**) Representative brightfield image of a culture which failed to produce 3-dimensional structures (EO-27). Asterisks (*) indicates $P < 0.05$.).
