# Supplementary material for: Patient-derived epithelial cell organoids mimic the phenotypic complexity of endometriosis subtypes
Source: Hum Reprod. 2025 Nov 27;41(2):262–74. doi: 10.1093/humrep/deaf230 (PMC12864149; doi:10.1093/humrep/deaf230)
Supplement: deaf230_Supplementary_Figure_S2 [file deaf230_supplementary_figure_s2.pdf]

Human Reproduction, 2025, 1–1
https://doi.org/10.1093/humrep/deaf230
Supplementary Data

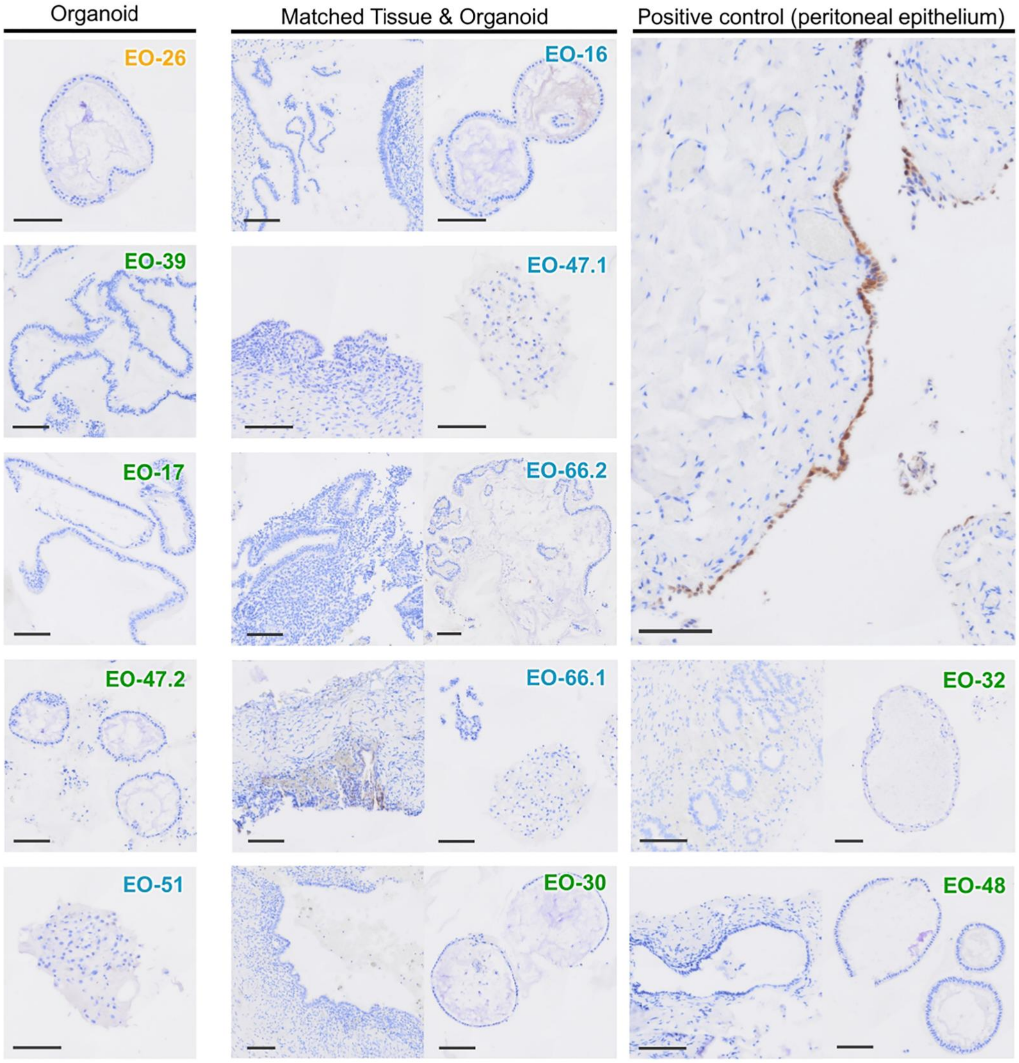

Supplementary Figure S2. Organoids recapitulate endometriosis lesion histology. Images show organoid models (first column, top to bottom: EO-26 [SUP, yellow], EO-39, EO-17, EO-47.2 [DIE, green], EO-51 [OMA, blue]) and matched tissue/organoid pairs (second and third columns, left to right, top to bottom: EO-16, EO-47.1, EO-66.2, EO-66.1 [OMA, blue], EO-30, EO-32, EO-48 [DIE, green]). Top right corner shows positive control for calretinin staining in peritoneal surface epithelium. Scale bars=200 μm.
