# Supplementary material for: Patient-derived epithelial cell organoids mimic the phenotypic complexity of endometriosis subtypes
Source: Hum Reprod. 2025 Nov 27;41(2):262–74. doi: 10.1093/humrep/deaf230 (PMC12864149; doi:10.1093/humrep/deaf230)
Supplement: deaf230_Supplementary_Figure_S3 [file deaf230_supplementary_figure_s3.pdf]

OXFORD
human reproduction

Human Reproduction, 2025, 1–1
https://doi.org/10.1093/humrep/deaf230
Supplementary Data

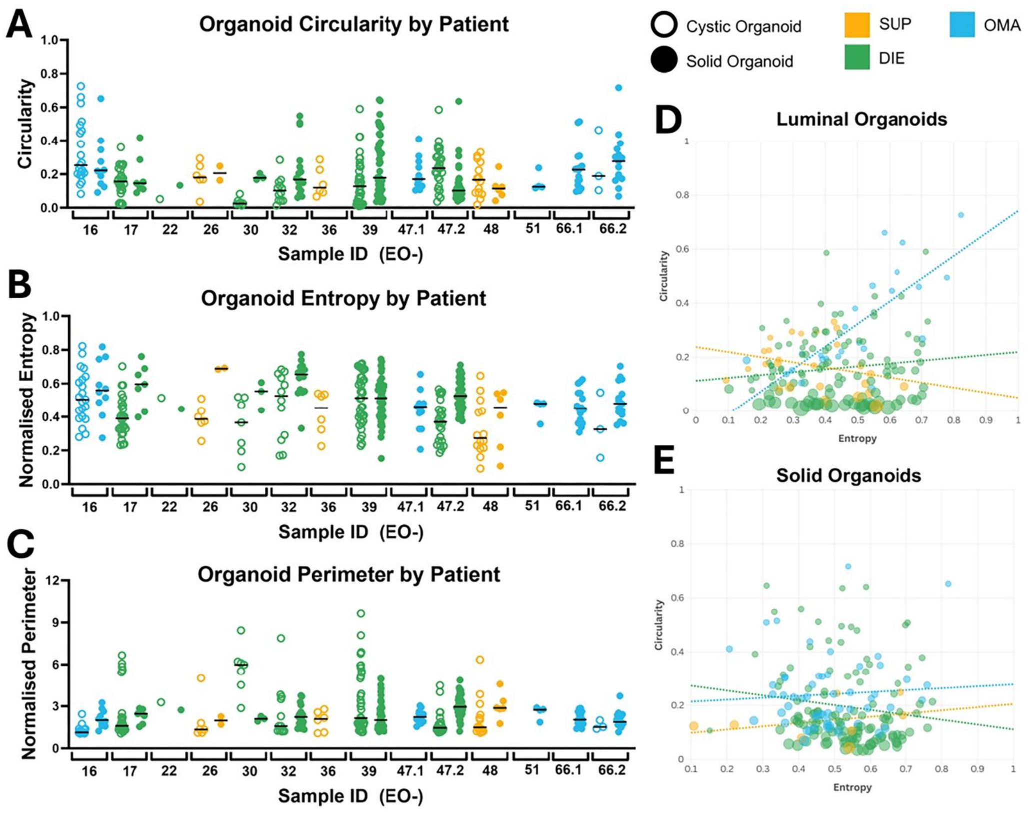

Supplementary Figure S3. Organoids develop cystic and solid morphologies, with quantifiable characteristics. (**A**) Organoid circularity by patient, separated by cystic and solid morphologies. (**B**) Organoid entropy by patient. (**C**) Organoid perimeter by patient. (**D**) Luminal organoids by circularity vs entropy. Size of each point proportional to organoid perimeter. Each data point represents one organoid. (**E**) Solid organoids by circularity vs entropy. Size of each point proportional to organoid perimeter. Each data point represents one organoid Colour corresponds to disease phenotype, where yellow=superficial endometriosis (SUP), green=deep infiltrating endometriosis (DIE), blue=endometrioma (OMA).
