# Supplementary material for: Patient-derived epithelial cell organoids mimic the phenotypic complexity of endometriosis subtypes
Source: Hum Reprod. 2025 Nov 27;41(2):262–74. doi: 10.1093/humrep/deaf230 (PMC12864149; doi:10.1093/humrep/deaf230)
Supplement: deaf230_Supplementary_Table_S1 [file deaf230_supplementary_table_s1.pdf]

OXFORD
human reproduction

Human Reproduction, 2025, 1–1
https://doi.org/10.1093/humrep/deaf230
Supplementary Data

Supplementary Table S1. Organoid media recipe.

Product	Supplier	Cat No.	Final Conc.
**DMEM/F12 advanced**	Thermo	12634010	
**HEPES**	Thermo	15630080	10 mM
**Glutamax**	Life Tech	35050061	1%
**N2**	Life Tech	17502048	1%
**B27 minus VA**	Thermo	12587001	1%
**bFGF**	R&D Systems	233-FB-010	2 ng/ml
**FGF-10**	Peprotech	100-26-5ugx2	10 ng/ml
**hEGF**	Sigma Aldrich	E9644	50 ng/ml
**p38i (SB202190)**	Sigma Aldrich	S7067-25mg	10 µM
**Nicotinamide (NICO)**	Sigma Aldrich	N0636-100G	10 mM
**A83-01**	Sigma Aldrich	SML0788-5MG	500 nM
**N-acetyl L-cysteine (NAC)**	Sigma Aldrich	A9165	1.25 mM
**hRSPO1**	Peprotech	120-38-20ug	50 ng/ml
**hNoggin**	Peprotech	120-10C	100 ng/ml
**β Oestradiol**	Sigma Aldrich	E2758	1 nM
**Insulin transferrin selenium**	Life Tech	41400045	2%
