# Supplementary material for: Patient-derived epithelial cell organoids mimic the phenotypic complexity of endometriosis subtypes
Source: Hum Reprod. 2025 Nov 27;41(2):262–74. doi: 10.1093/humrep/deaf230 (PMC12864149; doi:10.1093/humrep/deaf230)
Supplement: deaf230_Supplementary_Table_S2 [file deaf230_supplementary_table_s2.pdf]

OXFORD
human reproduction

Human Reproduction, 2025, 1–1
https://doi.org/10.1093/humrep/deaf230
Supplementary Data

Supplementary Table S2. Segmentation approaches for organoid shape analysis.

	Segmentation approaches
1	Using the green channel of the RGB image. Enhance contrast using the equalize_adapthist function (scikit-image package). Segmentation using a global thresholding algorithm (mean, Otsu) and removal of all foreground objects but the largest.
2	Convert to Haematoxylin-Eosin-DAB (HED) space and use the E channel. Segment the image using a global thresholding algorithm (minimum, Yen, Li or isodata) and invert it. Remove objects and holes with size smaller than an appropriate threshold (100, 200, 500, 1000, 2000 pixels). In most cases, the threshold for both the holes and the objects was the same.
3	Convert to HED space and use the D channel. Segment the image using a global thresholding algorithm (Yen, Otsu, or Li) and invert it. Apply a binary closing using an appropriate footprint (square of radius 3, 7 or 15 pixels; a diamond of side 5 pixels). Remove holes with area below a certain threshold (500, 2000 pixels) and objects smaller than a specified number of pixels (10, 1000 pixels). If necessary, manually select the segmented regions that are part of the organoid and remove the others. If necessary, apply a binary closing using an appropriate footprint (square of radius 7 or 19 pixels) and remove small holes (size <500 pixels).
4	Convert to HED space and use the D or E channel. Segment the image using a global thresholding method (Yen, minimum or Li) and invert it. Optionally remove holes and objects with area below a certain threshold (100 pixels). Apply a binary closing using an appropriate footprint (disk or square of radius 5 or 9 pixels).
5	Convert to HED space and use the H or E channel. Segment the image using a global thresholding method (Li, minimum) and invert it. Apply a binary closing using an appropriate footprint (square of side 7 pixels or a disk of radius 5, 7 or 11 pixels). Remove holes and objects with area below a certain threshold (300, 1000, 5000, 10 000, 13 000 pixels).
6	Convert to HED space and use the E channel. Segment the image using a global thresholding algorithm (Otsu) and invert it. Optionally remove small objects (area below 200 pixels). Manually select the segmented regions that are part of the organoid and remove the others. Remove holes with area below an appropriate threshold (5000 pixels). If necessary, apply a binary closing using an appropriate footprint (square of side 5 pixels).

Summary of the approaches used to segment the organoids. Most images were analysed using (1), with the segmentation algorithm chosen according to the organoid type. Some images required a different approach (2-6). The one best suited for each image was determined empirically, together with the relevant parameters (examples between brackets).

© The Author(s) 2025. Published by Oxford University Press on behalf of European Society of Human Reproduction and Embryology.
This is an Open Access article distributed under the terms of the Creative Commons Attribution License (https://creativecommons.org/licenses/by/4.0/), which permits unrestricted reuse, distribution, and reproduction in any medium, provided the original work is properly cited.
Human Reproduction, 2025, 00, 1–13
https://doi.org/10.1093/humrep/deaf230
Original Article
